# The Role of CTLA4 and Its Polymorphisms in Solid Organ and Haematopoietic Stem Cell Transplantation

**DOI:** 10.3390/ijms22063081

**Published:** 2021-03-17

**Authors:** Jakub Rosik, Bartosz Szostak, Filip Machaj, Andrzej Pawlik

**Affiliations:** Pomeranian Medical University, Department of Physiology, al. Powstańców Wielkopolskich 72, 70-111 Szczecin, Poland; jakubrosikjr@gmail.com (J.R.); bartszost1@gmail.com (B.S.); machajf@gmail.com (F.M.)

**Keywords:** CTLA-4, AHSCT, kidney transplantation, liver transplantation, GVHD

## Abstract

HLA matching, transplantation technique, or underlying disease greatly influences the probability of long-term transplantation success. It has been hypothesised that genetic variation affecting antigen presentation also contributes to the outcomes of both solid organ transplantation and allogeneic haematopoietic stem cell transplantation (AHSCT). Those genes, along with those responsible for innate and adaptive immunity, have become targets of investigation. In this review, we focus on the role of CTLA4 in the process of acute graft rejection and summarise the progress in our understanding of its role in predicting the outcome. We present the results of the latest studies investigating the link between *CTLA4* gene variability and AHSCT, as well as organ transplantation outcomes. While some studies found a link between +49 A/G and −318 C/T and transplantation outcomes, comprehensive meta-analyses have failed to present any association. The most recent field reviews suggest that the −1772 T/C (rs733618) CC genotype is weakly associated with a lower risk of acute graft rejection, while +49 A/G might be clinically meaningful when investigated in the context of combinations with other polymorphisms. Studies verifying associations between 12 CTLA4 gene SNPs and AHSCT outcomes present inexplicit results. Some of the most commonly studied polymorphisms in this context include +49 A/G (rs231775) and CT60 A/G (rs3087243). The results signify that, in order to understand the role of CTLA4 and its gene polymorphisms in transplantology, further studies must be conducted.

## 1. Introduction

Transplantology is a branch of medicine that has invoked strong feelings of both scientists and laymen for hundreds of years. The first successful transplantations of organs were great worldwide reported events. Currently, over 100,000 solid organ transplantations are conducted yearly, involving patients from more than 100 countries worldwide [1]. While surgical techniques have already been mastered, immunological side effects of transplantation and organ injuries induced by immunosuppression [2] are still a significant problem yet to be combated [3]. Moreover, some rarer serious complications involve specific groups of patients, such as children [4,5,6]. Recipients of dual transplantations might be an even greater challenge for doctors [7,8]. Those challenges indicate that there is an immense demand for progress in the field of transplantology [3]. After a few years of dynamic development of immunosuppressive agents [9], followed by a significant prolongation of graft survival, relatively little change has been observed in the last 30 years [6,10].

Currently, proteins participating in antigen presentation are under close scrutiny [3,11,12]. Single nucleotide polymorphisms (SNPs) in their genes might be vital in mediating the response of T cells to previously unknown antigens. A better understanding of the processes of chronic rejection and its underlying factors might not only prolong graft survival but also reduce the burden of immunosuppressive regimens and associated complications [3,13,14,15].

Allogenic haematopoietic stem cell transplantation (AHSCT) is a procedure aiming to treat a variety of haematological disorders, both malignant and non-malignant. The diseases most commonly treated with AHSCT are acute myeloid leukaemia (AML) or acute lymphoblastic leukaemia (ALL). Moreover, aplastic anaemia, immune deficiencies, and thalassaemia are non-malignant disorders potentially treatable with AHSCT [16,17]. Although highly efficacious, this method is not free of complications. Graft versus host disease (GVHD), a complex allogenic immune-mediated condition, is the leading cause of post-transplant mortality and morbidity [18,19,20]. Presentation of antigens in the context of HLA is the pivotal mechanism in the development of GVHD [21,22,23,24,25]. Recipient antigen-presenting cells (APCs), in particular dendritic cells, present recipient antigens unknown to donor lymphocytes. The second signal, a non-specific interaction between CD28 and CD80 or CD86 (B7.1 or B7.2), is susceptible to the inhibitory influence of CTLA-4. A process of antigen presentation similar to GVHD is hypothesised to play a central role in the antineoplastic effect of AHSCT—graft versus tumour (GVT) or graft versus leukaemia (GVL) effect [26]. In these processes, donor immune cells are able to selectively destroy tissue, leading to remission. Umbilical cord blood is an alternative to a bone marrow source of haematopoietic stem cells. The main advantage of cells acquired from the placenta and the umbilical cord is a much higher concentration of cells compared to adult blood. Nevertheless, even such high concentrations do not compensate for the low volume of umbilical cord blood [27]. Therefore, transplantation is often performed with cells originating from two donors [28]. Another advantage of cord blood transplantation (CBT) is T-lymphocyte immaturity [29]. Thus, GVHD does not follow CBT as often as typical AHSCT [27].

**Figure 1:**
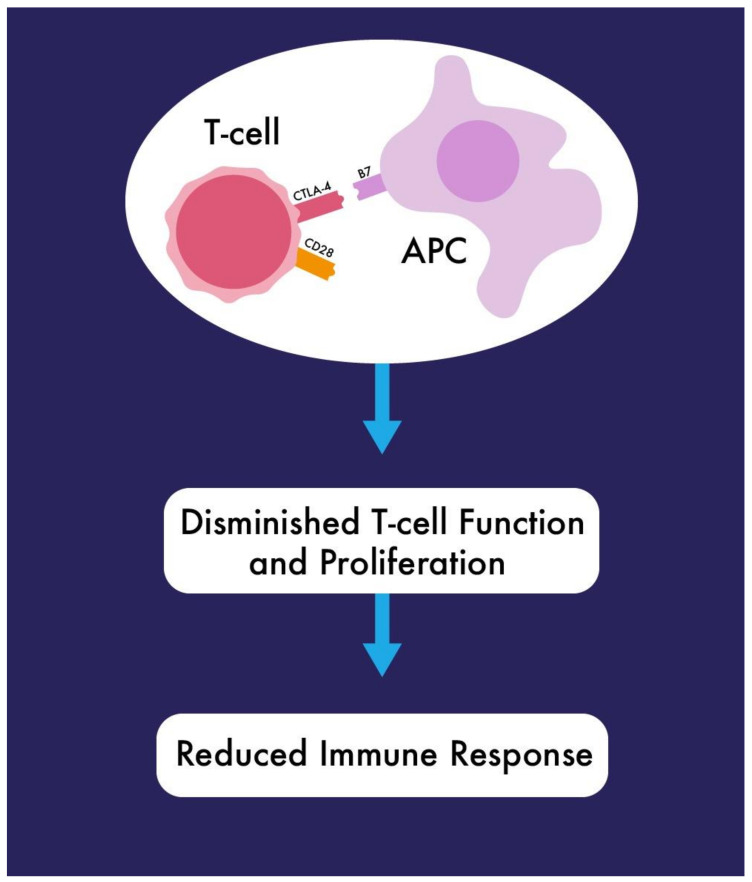
CTLA4 binds to B7.1 and B7.2 with higher avidity than CD28, leading to the reduction of IL-2 production and inhibition of T-cell activation.

CTLA4 is a glycoprotein expressed on the surface of T-lymphocytes [30,31]. While it has a regulatory function in the beginning stages of cell activation, its expression level is usually relatively low [32]. It exhibits its inhibitory influence on T-cell activation by blocking the second signal of activation from APCs [33]. As it has higher avidity for B7.1 and B7.2 [31,33] binding, it is able to disengage the signal conducted by CD28, resulting in decreased interleukin (IL)-2 production [34,35]. Prolonged negative signalling diminishes T-cell function and proliferation [36]. The CTLA4-mediated response involves both enhanced T-regulatory cell activity and decreased T-helper cell activity [32,34,37]. A short summary of the mechanisms of action of CTLA4 is presented in Figure 1.

As CTLA4 promotes the suppression of the immune system, its high expression can lead to faster progression or even to the development of neoplasms. Its overexpression has been identified in melanoma [32,38] and B-cell chronic lymphocytic leukaemia [39,40].

Located on chromosome 2, *CTLA4* gene polymorphisms might play an influential role in the development of autoimmune diseases and neoplasms [41,42]. Out of more than 100 *CTLA4* gene polymorphisms [31], especially rs3087243, which might influence the efficacy of alternative splicing [43], rs231775, influencing the amino acid at position 17, as well as CTLA4 surface expression [44] and rs5742909, associated with promoter activity [45], are most often the target of the studies. In particular, rs231775 seems to be an interesting research target. Its influence on CTLA-4 expression possibly determines the risk of autoimmune disease and cancers [46,47,48]. A study by Ramzi et al. showed that patients with AML have significantly higher levels of CTLA4 mRNA in peripheral blood lymphocytes compared to those in healthy controls [49]. The overexpression of CTLA4 on T cells could be the reason for their defective functioning and increased immunosuppression. Furthermore, the immunosuppression could be enhanced by the elevated levels of CTLA4 on surface of Tregs [50], acting in favour of disease progression [49]. 

**Figure 2:**
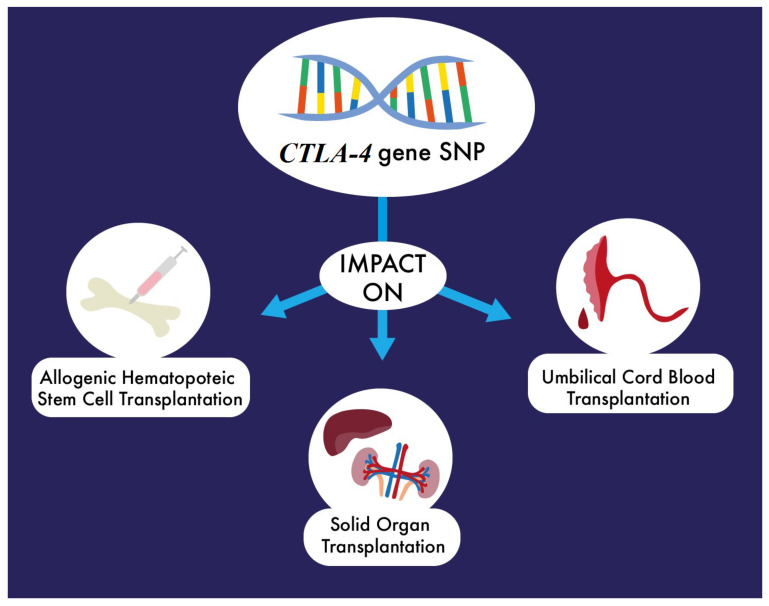
CTLA4 is an important immunomodulatory protein and its genetic variations are involved in the pathogenesis of various diseases. *CTLA4* gene SNPs are suspected to influence outcomes of different types of transplantation, such as allogeneic haematopoietic stem cell transplantation, umbilical cord blood transplantation, and solid organ transplantations (kidney and liver transplantations).

Genetic variations affecting expression of CTLA4 are known to influence the immune response, resulting in a higher risk for autoimmune and neoplastic diseases. The associations between them and transplantation outcomes have recently been verified. The types of transplantation that could be possibly influenced by *CTLA4* gene polymorphisms are presented in Figure 2. 

## 2. *CTLA4* Gene Polymorphisms and Solid Organ Transplantation Outcomes

A limited number of studies have investigated the effects of *CTLA4* gene polymorphisms on liver and kidney transplantation outcomes (Table 1). The mechanistic rationale of involvement of CTLA4 has been described by Minguela et al., who suggested that the expression of B7 and CD28/CTLA4 on peripheral lymphocytes contribute to graft acceptance or rejection [51]. The expression of co-stimulatory molecules was significantly elevated in the acute rejection group [51]. These findings indicate that *CTLA4* gene polymorphisms could affect the immune response and thereby alter the outcome of transplantations.

Perhaps the most emphasis has been placed on identifying the associations between acute rejection and two common SNPs within the *CTLA4* gene (+49 A/G (rs231775) and −318 G/T). A univariate analysis revealed a weak association of recipient *CTLA4* −318 G/T and +49 A/G genotype distributions with acute rejection. Only the *CTLA4* +49 SNP, but not the −318 SNP, was associated with acute rejection; this association was independent of other risk factors. Liver transplant recipients carrying either *CTLA4* +49 A/A or A/G had an 8.7 times greater relative risk of acute rejection compared with G homozygotes (OR = 8.7; 95% CI = 1.2–100; *p* = 0.02) [52].

A multivariate analysis revealed the rs231775 *CTLA4* gene G allele is an independent factor associated with an increased risk of delayed graft function (DGF) of the kidney (*p* = 0.03). A weak association was identified, showing that the frequency of DGF was higher among patients with the G allele of rs231775 (GG+AG vs. AA, OR = 1.80; 95% CI = 1.02–3.18; *p* = 0.05) [53].

In contrast, multiple single-centre studies have failed to identify the association of both +49 A/G and −318 C/T with acute graft rejection [44,54,55].

The associations have further been disproved by two comprehensive, independent meta-analyses, which both had over 2000 cases analysed; one investigated both +49 and −318 (2081 cases), and the other focused solely on the −318 polymorphism (2356 cases) [56,57].

The most recent meta-analysis by Cargnin et al. collected data from an even broader group of patients. This study included 5401 kidney transplant recipients combined from 15 cohorts. No association between +49 A/G and the risk of acute renal graft rejection was reported; neither of the alleles influenced the frequency of this complication [58]. The authors of the study excluded the influence of this SNP on acute graft rejection. Nevertheless, it is plausible that +49 A/G, when in combination with other genetic variants, might still determine the outcome of transplantation. Verification of hypothetical associations between graft survival and combinations of the most common SNPs is a potential new research direction. When describing the limitations of the meta-analysis, Cargnin et al. mention that large-scale studies on Asian or African populations might lead to other results than those amongst Caucasians [58].

Another field synopsis by Cargnin et al. investigated the relationship between gene polymorphisms and risk of acute graft rejection; a cumulative assessment of two studies investigating *CTLA-4* gene −1772 T/C polymorphism was performed. The results were borderline significant—frequency of graft rejection was lower in patients with the CC genotype (CC vs. TT, OR = 0.32; 95% CI = 0.11–0.97; *p* = 0.044) [59].

Microsatellite (ATn) repeat polymorphisms have been identified as one factor related to the increased incidence of acute rejection in association with alleles 3 and 4 in both liver and kidney transplantations (*p* = 0.002 and *p* = 0.05, respectively). Allele 7 was associated with acute rejection (*p* < 0.05), and a potential protective effect of allele 1 was suggested (*p* = 0.058) [54]. While Niknam et al. have identified the *CTLA4* gene 1661 SNP as a potential sex-dependent risk factor for the development of acute rejection [60], a subsequent comprehensive review argues that the connection is not that obvious and that the study failed to demonstrate a connection between acute rejection and the 1661 SNP, as well as the −1772 and −1147 *CTLA4* gene SNPs [61].

Although the G allele at the CT60 (SNP 3087243; +6230GNA) position was associated with a 50% decrease in soluble CTLA4 isoforms [62], a single study by Azarpira and colleagues failed to identify a connection between CT60 variants and acute rejection after liver transplantation [63].

**Table 1:** The association between common recipient SNPs within the *CTLA-4* gene and acute graft rejection in solid organ transplantation.

Author	Polymorphism	Association with acute graft rejection
Karimi et al. [55]	−318 C/T (rs5742909)	No association
Karimi et al. [55]	+49 A/G (rs231775)	A allele OR = 2.93; 95% CI = 0.94–10.24; *p* = 0.04
Karimi et al. [55]	−1661 A/G (rs4553808)	AA OR = 0.34; 95% CI = 0.91–1.09; *p* = 0.037 AG OR = 2.9; 95% CI = 0.92–9.16; *p* = 0.037 A allele OR = 6.47; 95% CI = 0.13–1.11; *p* = 0.043
Jiang et al. [44]	+49 A/G (rs231775)	No association
Zhu et al. * [56]	+49 A/G (rs231775)	No association
Zhu et al. * [56]	−318 C/T (rs5742909)	No association
Cargnin et al. * [58]	+49 A/G (rs231775)	No association
Cargnin et al. * [59]	−1772 T/C (rs733618)	CC genotype associated with lower risk of acute graft rejection (CC vs. TT, OR = 0.32; 95% CI = 0.11–0.97; *p* = 0.044)
Yang et al. * [57]	−318 C/T (rs5742909)	No association in overall population, T allele associated with risk of acute rejection in African population
Niknam et al. [60]	−1661 A/G (rs4553808)	AA genotype and A allele with low frequency in males in the acute rejection group (*p* = 0.03 and *p* = 0.04, respectively)
Han et al. * [61]	−1772 T/C (rs733618)	No association
Han et al. * [61]	−1661 A/G (rs4553808)	No association
Han et al. * [61]	−1147 C/T (rs16840252)	No association
Azarpira et al. [63]	CT60 A/G (rs3087243)	No association

* - meta-analysis.

## 3. The Role of CTLA4 and Its Polymorphisms in Allogeneic Haematopoietic Stem Cell Transplantation on the Influence of the *CTLA4* Gene on the Health of Patients after AHSCT

A study on 123 patients after AHSCT proved that both donor and recipient *CTLA4* gene polymorphisms could influence the outcome of transplantations. In a multivariate analysis, the *CTLA4* gene −318 TT genotype increased the risk of disease relapse. This polymorphism was not connected with the frequency of acute or chronic GVHD [64].

Research conducted on 72 patients with thalassaemia after AHSCT identified a polymorphism that reduces the risk of acute GVHD. Acute GVHD grade 2–4 occurred more often in patients with CT60 AA than in patients carrying genotype AG or GG [65]. In another study on 312 patients, a GG genotype in recipients was found to be a risk factor for acute GVHD [66]. The influence of the same polymorphism was verified by Mossallam and Samra in a study with 94 patients. The researchers did not find any association between donor or recipient genotype and clinical outcomes [67]. Mossallam and Samra checked the influence of +49 A/G on 80 patients and found the following association: recipients with the G allele had lower survival indicators than people with the AA genotype [67]. Another association concerning +49 A/G was found in a group of 152 patients, where the donor GG genotype was a risk factor for lower overall survival [68].

Contradictory results were reported by Piccioli et al., who found that, in a multivariate analysis, the +49 A/G, GG genotype in a recipient was a factor that prolonged overall survival [69].

The +49 A/G influence was further verified in a paediatric cohort of 88 patients. In a multivariate study, the AA genotype in donors was an independent risk factor for relapse [70]. This genotype was also associated with worse event-free survival [70]. In another paediatric cohort, the donor’s CT60 GG genotype led to lower transplant-related mortality risk and better event-free survival [71].

Based on a study on 240 patients, the CT60 AA donor’s genotype was associated with lower rates of grade II–IV acute GVHD in a group of transplants from unrelated donors [72]. In the same analysis, this genotype was associated with more relapses and early cytomegalovirus infections. The researchers hypothesised that this polymorphism may be important for T-cell reactivity [72].

Research on a cohort of 164 patients was set with the task to compare the influences of several *CTLA4* gene polymorphisms (rs11571315, rs11571316, rs16840252, rs231775, rs231777, rs231779, rs3087243, rs1019701, rs231725 and rs231775) on clinical outcomes; none of them were significantly connected with either overall survival or relapse-free survival. Results on the influence of rs4553808 were inexplicit [73].

Pérez-García et al. conducted research on donors of 536 HLA-identical AHSCTs. In a multivariate analysis, no significant connections were found for the +49 AG polymorphism. Moreover, the −1722 TC, −1661 AG and −318 CT polymorphisms were clinically insignificant. However, the G allele in position CT60 was a negative predictor of 5-year survival (56.2% vs. 69.8%), although acute GVHD occurred more frequently when the genotype of the donor was CT60AA [43].

It is vital to adduce an analysis with 80% power to detect any association between the 10 *CTLA4* gene SNPs and clinical outcomes of donors [74]. This finding undermines the theory of the potential influence of single variations in the *CTLA4* gene structure of donors and the effect on AHSCT.

Some studies have aimed to search for combinations of polymorphisms that influence the clinical outcomes. In a Japanese population, the donor haplotype of −318C/49A/CT60A was connected with a lower risk of disease relapse [75]. A similar study on 112 genotyped Tunisian patients with acute or chronic grade 0–IV GVHD was performed. A CTLA4 −318C/+49G nucleotide combination in donors was associated with a higher occurrence of chronic GVHD [76]. Only the 49G allele, which, according to Saadi et al., is likely associated with CMV infection [77], might be a predictor for chronic GVHD [76]. Detailed information about the results of these studies is presented in Table 2.

It is difficult to foresee whether any CTLA4 polymorphism will become routinely verified before transplantation in order to improve donor and recipient selection. The results for rs3087243 are inexplicit, but the probable influence of any of these alleles is clinically insignificant. 

The expression of CTLA-4 could be influenced by SNPs within its gene, such as rs231775. However, when examining CTLA-4 levels, Ramzi et al. found only a non-significant decrease in its expression in patients with AML who were diagnosed with aGVHD following AHSCT [49]. 

SNPs in genes important for T-cell function might be vital not only for bone marrow transplantation but also CBT. Our research group has summarised the available publications on the role of *CTLA4* gene polymorphisms in CBT outcomes. So far, the preliminary data present in the field is insufficient to draw final conclusions. In the future, a potential association between the rs3087243 GG genotype of the *CTLA4* gene polymorphism and CBT results should be verified [27].

So far, several studies have examined the associations between CTLA4 and its polymorphisms and the outcomes of solid organ and haematopoietic stem cell transplantation. Unfortunately, the results of these studies are inconclusive and differ among various populations. These differences may be due to linkage between CTLA4 gene polymorphisms and polymorphisms in other genes influencing the immune response after transplantation, such as T-cell activation pathways, signal transducers and transcriptional activators, as well as genes encoding mediators involved in the inflammatory response, such as cytokines and chemokines. Moreover the studies used various diagnostic criteria for post-transplant complications and immunosuppressive treatment regimens, included different numbers of subjects, and had different statistical power. Numerous genetic and immunological factors influence the outcome of transplantation. *CTLA4* gene polymorphisms may be just one of the many factors that increase the risk of developing certain complications. Therefore, they should be considered together with other known factors influencing the outcome of transplantation. It is important to identify the differences in various clinical parameters between genotypes that may affect transplantation outcomes. This approach will identify potential confounders that need to be included in a multivariate model for a specific gene polymorphism to be associated with graft loss. Gene polymorphisms may be taken into account as factors influencing the outcome of transplantation and increasing the risk of some post-transplant complications only together with other factors affecting these processes. The knowledge on the role of *CTLA4* gene polymorphisms in solid organ and haematopoietic stem cell transplantation is mainly based on candidate–gene association studies Candidate–gene association studies are prone to undetectable population differences. In contrast, genome-wide association studies (GWAS) have internal checks that address these concerns and therefore are in general much superior to candidate–gene studies in providing evidence for an association. Unless a variant shows significant association with a trait or disease in meta-analysis of several candidate–gene association studies, any single report from a candidate–gene association study must be interpreted cautiously as potential false positive finding. In order to know the exact role *CTLA4* gene polymorphisms in solid organ and haematopoietic stem cell transplantation, numerous multi-centre GWAS should be performed, which may confirm the role of selected CTLA4 polymorphisms in transplantology. 

## 4. Conclusions

Co-stimulation plays a crucial role in the activation of T cells, and blocking the co-stimulatory signals leads to T-cell anergy, resulting in better allograft tolerance. CTLA4 is expressed in T cells and inhibits the secondary signal activation from APCs. CTLA4 is one of the immunomodulatory molecules that is targeted in immunosuppressive therapy [78]. The fusion proteins belatacept and abatacept contain the extracellular domain of the CTLA4 protein and human Fc part of IgG [79,80], acting as soluble CTLA4, leading to a decrease in memory B-cell formation, production of antibodies, and T-cell responses [81,82]. These fusion proteins are used not only in the treatment of autoimmune diseases, such as rheumatoid arthritis, but also in the prophylaxis of kidney transplant rejection [80,83]. 

The *CTLA4* gene consists of three exons and two introns. More than 100 CTLA4 gene polymorphisms have been detected; some of these polymorphisms may have functional activity that changes the properties of CTLA4. Previous studies have indicated that *CTLA4* gene polymorphisms may be associated with the development of autoimmune diseases and cancers [37]. Therefore, CTLA4 became one of the targets in cancer therapy [84]. The blockade of CTLA4 attenuates its immunosuppressive effect, leading to sensitisation to tumour antigens [37]. The novel anti-CTLA4 agents have already been approved or are under examination in the context of treatment of melanoma, non-small cell lung cancer, oesophageal squamous cell carcinoma and renal cell carcinoma [85,86,87,88]. 

In solid organ transplantation, several studies have identified associations between *CTLA4* gene SNPs and transplantation outcomes. Namely, the A allele of +49 A/G (rs231775) and AA genotype of −1661 A/G (rs4554808) have been associated with higher rates of acute graft rejection, while the CC genotype of −1772 T/C (rs733618) has been associated with a lower risk of acute graft rejection. However, such results have not been, to date, replicated in larger scale meta-analyses. A 2020 meta-analysis by Cargnin et al. failed to identify any association between the +49 A/G (rs231775) polymorphism and transplantation outcomes [58], whereas Han et al. found no associations for −1661 A/G and −1772 T/C [61]. 

Genes that regulate the expression of proteins associated with T-cell activation are promising candidate genes for predicting transplantation results. In all, systemic reviews analysing results from meta-analyses and GWASs underline the fact that, thus far, none of the polymorphisms identified has strong epidemiological credibility. In the case of renal transplantation, out of the variants in *ACE*, *CD28*, *CTLA-4*, *CYP3A5*, *IFNG*, and *TNFα*, only TT/AT vs. AA of *IFNγ* +874 T/A reaches moderate epidemiological credibility, while the others can only be classified as weak evidence. Therefore, it is warranted to conduct further large-scale replication studies in order to identify the reliable genetic risk factors for acute renal and liver graft rejection.

In the case of AHSCT, the majority of the studies points to the recipient G allele of +49 A/G (rs231775) as a predictive factor for worse event-free survival and overall survival, while, on the contrary, Piccioli et al. have found that the GG recipient genotype is associated with longer overall survival. Similar discrepancies are present when scrutinising the donor +49 A/G (rs231775) polymorphism. Qin et al. have found that the GG donor genotype is associated with lower overall survival [68], while Hammrich et al. state that the AA donor genotype is associated with higher relapse rate and worse event-free survival [70]. Moreover, the G donor allele is believed to be associated with a higher risk of chronic GVHD; −318 C/T (rs5742909) has been investigated in one study to date, and a weak association has been found for the TT donor genotype and higher risk of disease relapse. *CTLA-4* gene SNP CT60 A/G (rs3087243) is perhaps the most studied out of all in the context of AHSCT outcomes. As far as recipient genotype is concerned, one dissertation has identified the AA genotype as the one associated with higher risk of acute GVHD, while another states the opposite—GG genotype is the predisposing one. Discrepancies in study results are also present in the case of donor genotype, as both the GG and AA genotypes have been found as indicative of a lower risk of mortality or incidence of GVHD.

Taken together, the results signify that, in order to understand the role of CTLA4 and its gene polymorphisms in transplantology, further studies, especially GWAS must be conducted. 

## Figures and Tables

**Table 2:** The effect of *CTLA-4* gene polymorphisms on allogenic haematopoietic stem cell transplantation outcomes, with regard to either donor or recipient polymorphisms.

Author	Polymorphism	Effect
Wu et al. [64]	−318 C/T (rs5742909)	TT donor genotype - higher risk of disease relapse - HR = 5.91 (1.17–29.79), *p* = 0.0313
Orrù et al. [65]	CT60 A/G (rs3087243)	AA recipient genotype - higher risk of acute GVHD: grade II–IV (63.2% vs. 24.5%), *p* = 0.001 and HR = 2.8 (1.2–6.6), *p* = 0.016; grade III–IV (36.4% vs. 7.6%); *p* = 0.005
Karabon et al. [66]	CT60 A/G (rs3087243)	GG recipient genotype - higher risk of acute GVHD - OR = 2.63 (1.45–4.59), *p* = 0.001
Mossallam et al. [67]	+49 A/G (rs231775)	G recipient allele - worse disease-free survival - HR = 2.17 (1.05–4.48), *p* = 0.03
Mossallam et al. [67]	+49 A/G (rs231775)	G recipient allele - worse overall survival - HR = 2.54 (1.16–5.54), *p* = 0.01
Qin et al. [68]	+49 A/G (rs231775)	GG donor genotype - lower overall survival - HR = 0.306 (0.111–0.842), *p* = 0.022
Piccioli et al. [69]	+49 A/G (rs231775)	GG recipient genotype - longer overall survival - *p* = 0.027
Hammrich et al. [70]	+49 A/G (rs231775)	AA donor genotype - higher relapse rate - 40% vs. 19%, *p* = 0.028
Hammrich et al. [70]	+49 A/G (rs231775)	AA donor genotype - worse event free survival - 46% vs. 70%, *p* = 0.025
Hammrich et al. [71]	CT60 A/G (rs3087243)	GG donor genotype - lower transplant-related mortality risk - HR = 0.507 (0.301–0.853), *p* = 0.011
Hammrich et al. [71]	CT60 A/G (rs3087243)	GG donor genotype - better event-free survival - HR = 0.704 (0.509–0.975) *p* = 0.035
Xiao et al. [72]	CT60 A/G (rs3087243)	AA donor genotype - lower incidence of grade II–IV acute GHVD (16.7% vs. 47.5%), *p* = 0.016
Xiao et al. [72]	CT60 A/G (rs3087243)	AA donor genotype - higher incidence of early CMV infections: transplantations from unrelated donor, 84.2 % vs. 59.7 %, *p* = 0.027; sibling transplantations, 90% vs. 41.3%, *p* < 0.0001
Xiao et al. [72]	CT60 A/G (rs3087243)	AA donor genotype - higher acute myeloid leukaemia relapse rate - RR = 2.792 (1.013–7.696), *p* = 0.047
Pérez-García et al. [43]	CT60 A/G (rs3087243)	G donor allele - worse overall survival - HR = 3.80 (1.75–8.22), *p* = 0.001
Pérez-García et al. [43]	CT60 A/G (rs3087243)	AA donor genotype - higher risk of acute GVHD - HR = 1.54 (1.03–2.29), *p* = 0.033
Sellami et al. [76]	+49 A/G (rs231775)	G donor allele - higher risk of chronic GVHD - OR = 2.58 (1.05–6.32), *p* = 0.032

## Data Availability

Not applicable.
